# Neuroprotective Effect and Molecular Mechanism of [6]-Gingerol against Scopolamine-Induced Amnesia in C57BL/6 Mice

**DOI:** 10.1155/2018/8941564

**Published:** 2018-01-31

**Authors:** Chang-Yul Kim, Yongtaek Seo, Chan Lee, Gyu Hwan Park, Jung-Hee Jang

**Affiliations:** ^1^Department of Pathology, College of Oriental Medicine, Daegu Haany University, Daegu 42158, Republic of Korea; ^2^Department of Pharmacology, School of Medicine, Keimyung University, Daegu 42601, Republic of Korea; ^3^College of Pharmacy, Research Institute of Pharmaceutical Sciences, Kyungpook National University, Daegu 41566, Republic of Korea

## Abstract

We have investigated the neuroprotective and memory enhancing effect of [6]-gingerol (GIN), a pungent ingredient of ginger, using an animal model of amnesia. To determine the neuroprotective effect of GIN on cognitive dysfunction, scopolamine (SCO, 1 mg/kg, i.p.) was injected into C57BL/6 mice, and a series of behavioral tests were conducted. SCO-induced behavior changes and memory impairments, such as decreased alteration (%) in Y-maze test, increased mean escape latency in water maze test, diminished step-through latency in passive avoidance test, and shortened freezing time in fear condition test, were significantly prevented and restored by the oral administration of GIN (10 or 25 mg/kg/day). To further verify the neuroprotective mechanism of GIN, we have focused on the brain-derived neurotrophic factor (BDNF). The administration of GIN elevated the protein expression of BDNF, which was mediated via the activation of protein kinase B/Akt- and cAMP-response element binding protein (CREB) signaling pathway. These results suggest that GIN may have preventive and/or therapeutic potentials in the management of memory deficit and cognitive impairment in mice with amnesia.

## 1. Introduction

Neurodegeneration in individuals with Alzheimer's disease (AD), Parkinson's disease (PD), and other neurodegenerative disorders is multifactorial, of which a complex set of toxic reactions, including inflammation, glutaminergic neurotoxicity, increased iron levels, accumulation of reactive oxygen and/or nitrogen species (ROS/RNS), depletion of endogenous antioxidant, reduced expression of neurotropic factors, and increased expression of apoptosis-related proteins, lead to neuronal cell death and damage [1]. Learning and memory are generated by an experience-dependent and long-lasting modification of the central nervous system (CNS), which involve the activation of diverse neurotransmitters, such as dopamine and serotonin, and the receptor-linked enzymes that are responsible for the activation of intercellular second messengers [2]. Particularly, cholinergic neurons in the brain of patients with AD are vulnerable, and cholinergic function plays an important role in the learning and memory process. Cholinergic neurons in the CNS are degenerated in patients with AD, which is correlated with senile dementia severity and degree of degeneration [3].

In this study, because the regulation of the cholinergic system is a key process in memory function, scopolamine (SCO), an experimental tool that induces cognitive dysfunction, was utilized, which is a tropane alkaloid exhibiting muscarinic antagonistic effects. The administration of SCO induces central cholinergic blockade and produces a reversible and well-described impairment in maintaining attention, processing information, and acquiring new knowledge in rodents and humans [4]. In* in vivo* animal models, SCO induced deficits in learning and memory as assessed by contextual and cued fear conditioning, inhibitory avoidance, and spatial learning tasks [4, 5]. Recently, studies showed that memory impairment in an SCO-treated animal model is associated with the altered status of brain oxidative stress and subsequent degenerative changes within the forebrain cholinergic system [6].

Biologically active phytochemicals in plants or plant products and herbal ingredients that can be used to develop practical natural medicines have been a topic of interest [7]. Traditional oriental herbs contain a wide variety of biologically active compounds, which is being used for the treatment of various types of chronic and acute diseases for thousands of years. Increasing evidence has suggested that traditional herbs can prevent and/or treat pathological outcomes from neurodegenerative disorders, and herbal medicine has been used by the general population because of these effects [8].

Ginger (*Zingiber officinale* Roscoe, Zingiberaceae) is utilized as a traditional oriental medicine that alleviates conditions such as headache, nausea, and colds [9]. It also has gastroprotective effects against motion sickness and slow-wave dysrhythmias [10]. Gingerols are pungent compounds in the rhizome of ginger. Under conditions with elevated temperature, gingerols can be converted into less pungent chemicals, such as shogaols and zingerone. Shogaols are not found in fresh ginger and are normally generated by the dehydration of gingerol during heating or long-term storage [11]. Particularly, gingerols have been found to possess diverse pharmacological and physiological activities, including antipyretic, analgesic, cardiotonic, antioxidant, and anti-inflammatory effects [11–14]. The combination of ginger extract and* Ginkgo biloba (G. biloba)* [15] or ginger and* Cyperus rotundus* [16] improves cognitive dysfunction and protects against oxidative stress in age-related animal models with dementia. However, the memory enhancing effect of ginger extract alone or its active ingredients has not been investigated and verified. Therefore, this study aimed to examine the effect of ginger extracts and [6]-gingerol (GIN) in C57BL/6 mice with SCO-induced learning and memory impairments and to elucidate the underlying molecular mechanisms.

## 2. Materials and Methods

### 2.1. Materials

GIN was purchased from Wako (Osaka, Japan), and SCO was obtained from Sigma-Aldrich (MO, USA). Anti-phospho-cAMP-response element binding protein (p-CREB) and anti-phosphoprotein kinase B/Akt (p-Akt) antibodies were obtained from Cell Signaling Technology (TX, USA). Anti-Akt and anti-brain-derived neurotrophic factor (BDNF) antibodies were provided by Santa Cruz Biotechnology (CA, USA), and anti-actin antibody and other chemical reagents were bought from Sigma-Aldrich. Fresh and dried ginger was purchased from Sejong pharmaceutical manufacturer (Daegu, Korea), and 600 g of fragmented ginger was extracted in 2.6 L of water at 70°C for 1.5 h. The supernatant was filtered, concentrated (Rotavapor R-220, Buchi, Switzerland), and freeze-dried to powder form (FreeZone 6, Labconco, MO, USA).

### 2.2. Animals and Drug Administration

Twelve-week-old male C57BL/6 mice were used in this study (20 ± 5 g; Charles River Orient Inc., Seongnam, Gyeonggi-do, Korea). They were kept in a room with the following conditions: temperature at 23 ± 1°C and a 12-h light and 12-h dark cycle (lights turned on at 06:00 am). Fresh and dried ginger extracts (FGE and DGE) and GIN were suspended in normal saline and 1% ethanol, respectively. The mice were pretreated orally with FGE (100 or 200 mg/kg), DGE (100 or 200 mg/kg), or GIN (10 or 25 mg/kg) for 3 days before starting memory tests and continuously administered the drug during behavior tests. All experimental procedure was conducted in accordance with the National Institutes of Health and Daegu Haany University guidelines for Laboratory Animals, which was approved by the Committee for the Care and Use of Laboratory Animals in the Daegu Haany University.

### 2.3. Y-Maze Task

Y-maze task is frequently used in monitoring spatial learning. Animals were allowed to learn alternation between arms based on their memory of the previously visited arms. The experimental apparatus consisted of a white-painted Y-maze that is made from acryl. Each arm of the Y-maze was 30 cm long, 14 cm high, and 8 cm wide, and it is positioned at an equal angle. Each mouse was placed at the end of one arm and allowed to move freely through the maze during an 8-min session. Spontaneous alteration behavior was defined as the consecutive entry into all three arms in overlapping triplet sets. During each trial, spontaneous alternations were recorded using the EthoVision System (Noldus, Wageningen, Netherlands). The percentage (%) of spontaneous alternation behavior was determined by dividing the total number of alternations by the total number of arm entries, subtracting 2, and then multiplying by 100 according to the following equation: % alternation = [(number of alternations)/(total number of arm entries − 2)] × 100. One hour before the test, mice were orally administrated with vehicle, FGE (100 or 200 mg/kg), DGE (100 or 200 mg/kg), or GIN (25 mg/kg), and 30 min later, the mice were injected with vehicle or SCO (1 mg/kg, i.p.). The Y-maze arms were thoroughly cleaned in between tests to remove residual odors.

### 2.4. Morris Water Maze Test

The Morris water maze test is a behavioral procedure designed to test spatial memory. In this experiment, a stainless circular pool (120 cm in diameter and 45 cm in height) with a featureless inner surface that is filled with white colored water (21 ± 1°C) was used. A white platform (30 cm in height and 10 cm in diameter) was placed in one of the pool quadrants 2 cm below the surface. Four external cues to the maze were visible from the pool and used by the mice for spatial memory. The position of the cues remained unchanged throughout the experiments. During the four consecutive training days, the mice were subjected to three trials per day. One hour before the test, the mice were treated with vehicle, FGE (200 mg/kg), DGE (100 mg/kg), or GIN (25 mg/kg), and 30 min before the test, the mice were injected with vehicle or SCO (1 mg/kg). For each training trial, the rodents were placed in the water facing the pool wall at one of the pool quadrants in a different order each day. The time interval between each trial was 10 min. When the mice located the platform, they remained on it for another 10 sec. If the mice did not find the platform within 120 sec, they were guided to and placed on it for 10 sec. During each trial, the time taken for the mice to find the platform was recorded using the EthoVision System (Noldus, Wageningen, Netherlands). On the 4th day after the final training trial, the mice were subjected to the probe test in which the platform was removed from the pool. They were allowed to swim to search for the platform for 90 sec, and the time spent to find the platform quadrant was monitored.

### 2.5. Passive Avoidance Task

The passive avoidance task is a fear-motivated avoidance task in which the mice learn to refrain from stepping through a door to a dark compartment that is safer but one in which they were previously punished. This test was carried out in identical illuminated and nonilluminated boxes (16 cm long, 10 cm high, and 11 cm wide) that are connected by guillotine door (Gemini Avoidance System, SD, USA). The floor of both compartments was composed of 2-mm stainless rods that are 1 cm apart. An hour prior to the behavioral test, the mice were treated with vehicle or GIN (10 or 25 mg/kg), and 30 min after, they were injected with vehicle or SCO (1 mg/kg). For the acquisition trials, the mice were initially placed in the illuminated compartment, and the door between the two compartments was opened 20 sec later. When the mice entered the dark compartment, the door automatically closed, and an electrical foot shock (0.5 mA, 5 sec) was delivered through the stainless-steel rods. Twelve hours after the acquisition trial, the mice were replaced in the illuminated compartment for the retention test. The time taken for a mouse to enter the nonilluminated compartment after opening the door was defined as a step-through latency for both acquisition and retention trials. The maximum step-through latency to enter the dark compartment was restricted to 300 sec.

### 2.6. Contextual Fear Conditioning Task

Contextual fear conditioning is the method by which organisms learn to fear new stimuli. It is a form of learning in which fear is associated with a particular neutral context or stimulus. One hour before this test, the mice were orally administrated with vehicle or GIN (25 mg/kg), and after 30 min, they were intraperitoneally injected with vehicle or SCO (1 mg/kg). During training, the mice were placed in the conditioning cage (26 cm long, 28 cm wide, and 15 cm high) for 60 sec for adaptation. Sound tone (68–80 dB) was delivered for 15 sec as a conditioned stimulus, and after the last 1 sec of the tone stimulus, an electrical foot shock (0.5 mA) was delivered for 1 sec. This procedure was repeated two times at 150-sec intervals. One day after the training test, the mice were placed in the conditioning cage, and the freezing response was continuously measured for 5 min, which is defined as the absence of mouse paw moving and breathing.

### 2.7. Immunohistochemistry

After completing the behavioral tests, the mice were anesthetized with Zoletil with lompun and perfused with 4% paraformaldehyde (PFA) in 0.1-M phosphate buffer saline (PBS). The brains were removed and postfixed with 4% PFA at 4°C overnight and then incubated in a series of 10%, 20%, and 30% sucrose solution for cryoprotection. In addition, they were embedded in OCT compound and quickly frozen using liquid nitrogen. The frozen brains were cut into 30 *μ*m-thick sections using a cryostat (Leica, Wetzlar, Germany), placed onto the slides, and then incubated with 1% Triton X-100 in PBS for 10 min at room temperature (RT). After washing, sections were incubated in 0.6% hydrogen peroxide (H_2_O_2_) in DDW for 30 min in the dark to remove endogenous peroxidase activity, washed twice with PBS, and further treated with 5% bovine serum albumin (BSA) in PBS for 60 min at RT, followed by incubation with anti-p-CREB antibody in PBS containing 3% BSA overnight at 4°C. The sections were rinsed with PBS, treated with biotinylated anti-rabbit secondary antibody for 60 min at RT, and then treated with avidin–biotin complex reagent (Santa Cruz Biotechnology) for 30 min. The sections were washed again with PBS and developed using 0.025% 3,3-diaminobenzidine (DAB) with 0.003% H_2_O_2_ (Vector Laboratories Inc., CA, USA) in PBS and images were visualized under a light microscope (Leica, Germany).

### 2.8. Western Blot Analysis

After the behavioral tests, the mice were anesthetized with Zoletil and lompun, and the cortical tissues were dissected for western blot analysis. The isolated cortex tissues were homogenized in RIPA buffer that contains 50 mM Tris HCl (pH 8.0), 150 mM NaCl, 1% Triton X-100, 1 mM EDTA, 10 mM NaF, 1 mM Na_3_VO_4_, and Complete Protease Inhibitor Cocktail Tablet (Roche Diagnostics, IN, USA). The protein concentrations of the samples were determined via the BCA protein assay (Pierce, IL, USA). The protein samples were separated on 12.5% SDS polyacrylamide gels and transferred to a polyvinylidene fluoride (PVDF) membrane (Pall Co., MI, USA) for further processing. After blocking with 0.05% Tween 20 in PBS (PBST) that contains 5% nonfat dried milk for 1 h at RT, the membranes were incubated with primary antibodies in PBS that contains 3% skim milk at 4°C overnight. After three times of washing with PBST, the blots were further reacted with horseradish peroxidase- (HRP-) conjugated anti-rabbit or anti-mouse secondary antibody (1 : 5000, Enzo Life Sciences Inc., NY, USA) in PBS containing 3% skim milk for 1 h. After washing with PBST for three more times, the specific bands were detected with enhanced chemiluminescence (ECL) western blotting detection reagent (Amersham Biosciences, NJ, USA) and visualized by using an ImageQuant LAS 4000 Multi Gauge software (Fujifilm, Tokyo, Japan).

### 2.9. Statistical Analysis

The data were expressed as means ± SEM, and a statistical analysis for multiple comparisons was performed using one-way ANOVA, followed by the Tukey test using the SPSS software (SPSS 12.0 KO for windows). A *p* value < 0.05 was considered statistically significant.

## 3. Results

### 3.1. Effects of Ginger Extracts on SCO-Induced Memory Loss in C57BL/6 Mice

**Figure 1:**
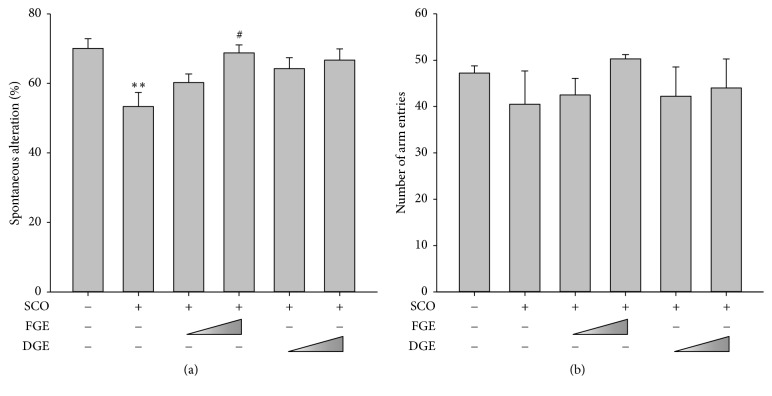
Memory enhancing effects of ginger extracts based on the Y-maze task. One hour before the test, the mice were treated with vehicle or ginger extracts (FGE, 100 or 200 mg/kg; DGE, 100 or 200 mg/kg; p.o.), and 30 min later, the mice were injected with vehicle or scopolamine (SCO, 1 mg/kg, i.p.). (a) Effects of ginger extracts on the SCO-induced spontaneous alternation. (b) Total number of arm entries drug during the 8-min trials of the Y-maze task. Data were presented as mean ± SEM (*n* = 4). Significant difference between the groups: ^*∗∗*^*p* < 0.01, vehicle-treated control versus SCO group; ^#^*p* < 0.05, SCO group versus ginger extract-treated group in combination with SCO.

Immediate working memory performance was assessed by recording spontaneous alteration behavior in a single session of a Y-maze task. In this test, SCO (1 mg/kg, i.p.) significantly decreased the percentage of spontaneous alteration (Figure 1(a)). The treatment of C57BL/6 mice with FGE (200 mg/kg) effectively improved memory impairment that is induced by SCO (Figure 1(a)). However, the total number of arm entries was similar in each experimental group (Figure 1(b)).

**Figure 2:**
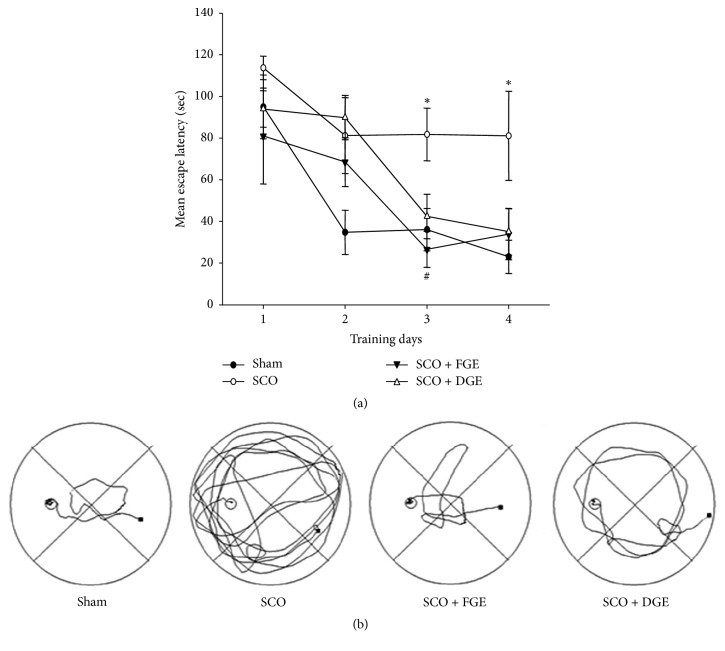
Cognitive enhancing effects of ginger extracts based on the Morris water maze test. One hour before the test, the mice were orally administered with vehicle or ginger extracts (FGE, 200 mg/kg; DGE, 100 mg/kg; p.o.), and after 30 min, the mice were injected with vehicle or SCO (1 mg/kg, i.p.). (a) Effects of ginger extracts on the SCO-induced spatial memory impairment that was represented by the mean escape latency. Data were presented as mean ± SEM (*n* = 4). Significant difference between the groups: ^*∗*^*p* < 0.05, vehicle-treated control versus SCO group; ^#^*p* < 0.05, SCO group versus ginger extract-treated group in combination with SCO. (b) The representative water maze paths that were selected from each group on the 3rd day of training trials.

In another experiment, the Morris water maze was conducted to compare spatial memory. The mean escape latency in finding the hidden platform during consecutive training days was calculated and compared. The mice in the vehicle-treated sham control group rapidly learned the hidden submerged platform, which they reached within 40 sec by the 3rd day of the training period (Figure 2(a)). In contrast, the performance of the SCO-treated group was significantly impaired. Repeated daily administration of FGE (200 mg/kg) reduced the mean escape latencies, particularly on the 3rd day (Figure 2(a)). The representative swimming-tracking paths of each group on the 3rd day are shown in Figure 2(b).

### 3.2. Restoration of SCO-Induced Learning and Memory Impairment due to the Use of GIN in the Passive Avoidance and Fear Conditioning Tests

**Figure 3:**
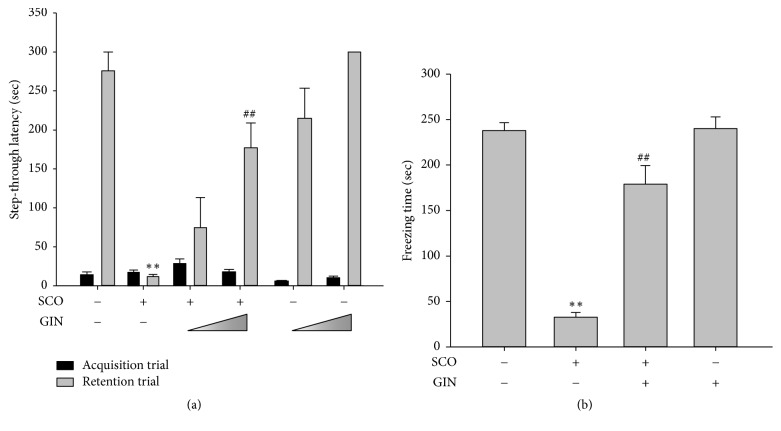
Protective effect of GIN on the learning and memory deficits caused by SCO based on the passive avoidance and contextual fear conditioning tests. One hour before this test, the mice were treated with vehicle or GIN (10 or 25 mg/kg, p.o.), and 30 min later, they were treated with vehicle or SCO (1 mg/kg, i.p.). (a) Effect of GIN on the SCO-diminished step-through latency was monitored during the retention trial. (b) Effect of GIN on the SCO-decreased immovable time without any movement for the freezing response was examined. Data were presented as mean ± SEM (*n* = 7). Significant difference between the groups: ^*∗∗*^*p* < 0.01, vehicle-treated control versus SCO group; ^##^*p* < 0.01, SCO alone group versus GIN-treated group in combination with SCO.

In the passive avoidance task, particularly during the acquisition trial, no significant difference was observed in the latency time among the experimental groups. In addition, no apparent difference in the vocalization of mice in each treatment group was observed when they received a foot shock. However, during retention trial, the step-through latency was significantly decreased in the SCO-treated group compared with the vehicle-treated control group. The SCO-reduced step-through latency was recovered by repeated oral administration of GIN with a dose of 25 mg/kg (Figure 3(a)).

In the contextual fear conditioning task, during the training trial, no significant difference was observed in each treatment group when they received a foot shock. However, after 24 h, a significant decrease in the freezing time was observed in the SCO-treated group compared with the vehicle-treated control group (Figure 3(b)). SCO effectively disrupted the conditioned fear to the context in C57BL/6 mice. Moreover, the freezing time was increased almost to control level by the repeated administration of GIN (25 mg/kg, p.o.) (Figure 3(b)).

### 3.3. Improvement in SCO-Induced Learning and Memory Deficit with the Use of GIN in the Y-Maze Task

**Figure 4:**
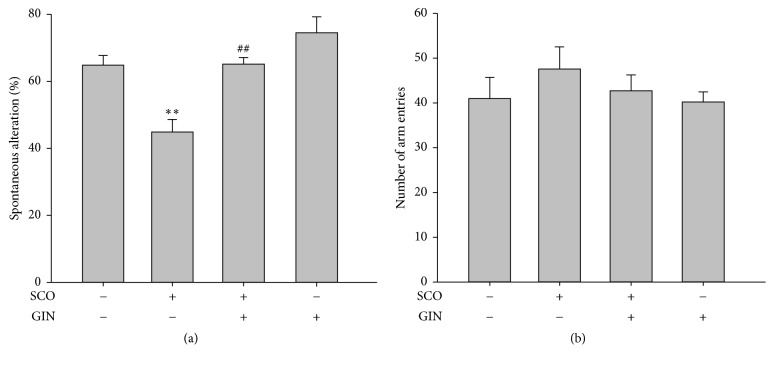
Protective effect of GIN on the learning and memory impairment induced by SCO in the Y-maze task. One hour before this test, mice were orally administered with vehicle or GIN (25 mg/kg, p.o.), and after 30 min, they were injected with vehicle or SCO (1 mg/kg, i.p.). (a) Effect of GIN on the SCO-decreased spontaneous alternation. (b) Total number of arm entries during the 8-min trials of the Y-maze task. Data were presented as mean ± SEM (*n* = 7). Significant difference between the groups: ^*∗∗*^*p* < 0.01, vehicle-treated control versus SCO group; ^##^*p* < 0.01, SCO group versus GIN-treated group in combination with SCO.

Immediate working memory performance was assessed by recording spontaneous alteration behavior in a single session of the Y-maze task. In the Y-maze task, the spontaneous alternation in SCO-treated mice was significantly lower than that of the vehicle-treated control mice (Figure 4(a)). The reduced alternation via i.p. injection of SCO was significantly reversed by the oral administration of GIN (Figure 4(a)). SCO slightly increased the total number of arm entries, which was not much affected by GIN treatment (Figure 4(b)).

### 3.4. Attenuation of SCO-Induced Cognitive Dysfunction with the Use of GIN in the Morris Water Maze Test

**Figure 5:**
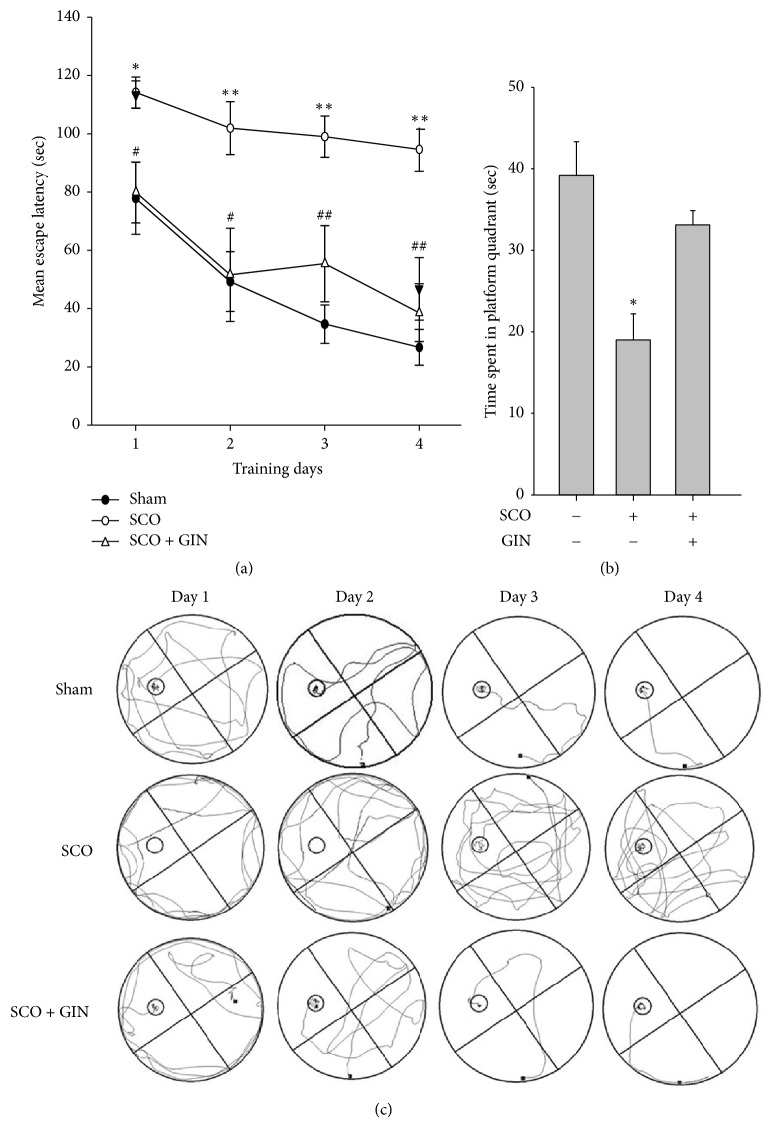
Protective effect of GIN on the SCO-induced cognitive dysfunction in the Morris water maze test. (a) Effect of GIN on the SCO-induced spatial memory impairment that was represented by the mean escape latency. (b) On the 4th day after the final training trials, the mice were subjected to probe trials in which the platform was removed from the pool, and they were allowed to swim for 90 sec to search for the platform. Data were presented as mean ± SEM (*n* = 7). Significant difference between the groups: ^*∗*^*p* < 0.05 and ^*∗∗*^*p* < 0.01, vehicle-treated control versus SCO (1 mg/kg, i.p.) group; ^#^*p* < 0.05 and ^##^*p* < 0.01, SCO alone group versus GIN-treated (25 mg/kg, p.o.) group in combination with SCO.

In the Morris water maze test, the SCO-treated group exhibited longer escape latencies, the time taken to find the platform, compared with the vehicle-treated control group throughout the training days. However, the oral administration of GIN (25 mg/kg) significantly reduced the mean escape latency, thereby ameliorating the learning and memory loss caused by SCO (Figure 5(a)). To further verify the spatial memory, the time spent in the platform quadrant during the probe trial was measured after the last training trial on the 4th day. Swimming times of the SCO-treated group within the platform quadrant were significantly lower than those of the vehicle-treated group (Figure 5(b)). The time spent reflects the retention of learning and memory. The sham and GIN-treated groups spent more time searching for the removed platform in the target quadrant than the SCO-treated amnesic group (Figure 5(b)).

### 3.5. Restoration of BDNF Expression with the Use of GIN Possibly through the Activation of Akt-CREB Pathway

**Figure 6:**
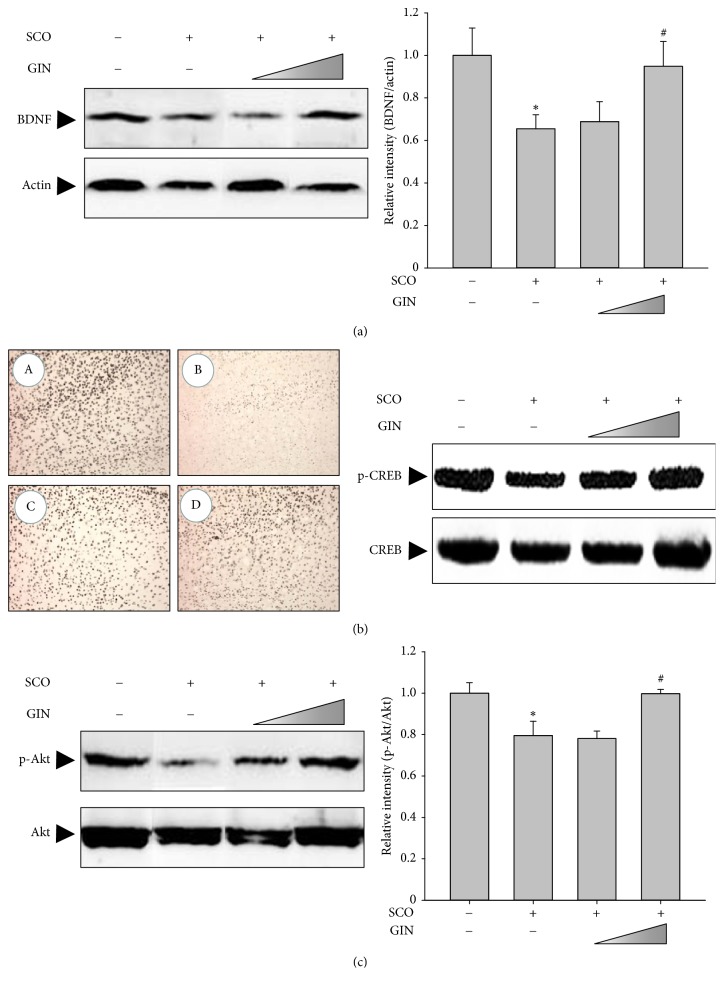
Effect of GIN on the SCO-reduced protein expression of BDNF in the cortex. Mice were pretreated with GIN for 3 days before the start of the test and then continuously administered during the behavior tests. The protein expression ratios of BDNF/actin (a) and p-Akt/Akt (c) were monitored via western blot analysis. Data were presented as mean ± SEM (*n* = 3). Significant difference between the groups: ^*∗*^*p* < 0.05, vehicle-treated control versus SCO group; ^#^*p* < 0.05, SCO alone group versus GIN-treated group in combination with SCO. (b) Effect of GIN on the activation of CREB via phosphorylation in the cortex was examined via immunohistochemistry by staining with anti-p-CREB antibody (right panel). (A) sham control, (B) SCO (1 mg/kg), (C) SCO (1 mg/kg) + GIN (10 mg/kg), (D) SCO (1 mg/kg) + GIN (25 mg/kg). The protein levels of p-CREB and CREB were also verified by western blot analysis (left panel).

To verify the neuroprotective mechanisms of GIN against SCO-induced deficits in learning and memory, the expression of BDNF and its upstream regulators were examined. The SCO-treated amnesic group exhibited decreased levels of BDNF protein in the cortex, which was increased by the oral administration of GIN (25 mg/kg) (Figure 6(a)). Moreover, the increased expression of BDNF seemed to be mediated by the activation of Akt-CREB signaling pathway. The amount of the phosphorylated form of CREB was remarkably reduced in the cortical tissues of the SCO-treated mice, and GIN treatment also increased the p-CREB levels in the SCO-treated group comparable to the sham control group (Figure 6(b)). Under the same experimental condition, the SCO-reduced relative ratio of p-Akt/Akt, a possible upstream kinase of CREB, was upregulated by GIN treatment (Figure 6(c)).

## 4. Discussion

As the average human lifespan has increased dramatically over the last decades, the human society has been more concerned with age-associated memory loss and neurodegenerative diseases, such as AD. Neurodegenerative disorders are a group of disease conditions that resulted from chronic breakdown and progressive functional and structural loss of the neurons due to neural damage and cell death. Naturally occurring phytochemicals can strongly develop biologically active compounds with antiamnesic and anti-AD activities. However, only some natural sources, such as* G. biloba *and* Huperzia serrata*, have been extensively studied because of their natural therapeutic properties to treat patients with AD. Therefore, in this study, we have investigated the neuroprotective and memory enhancing effects of ginger extracts and one of its major constituents (GIN) against SCO-induced cognitive dysfunction in C57BL/6 mice.

Increasing evidence from experimental as well as clinical studies clearly indicates the major roles of acetylcholine (ACh), choline acetyltransferase (ChAT), and acetylcholine esterase (AChE) in the regulation of cognitive functions [3]. Based on a cholinergic hypothesis, many attempts have been made to reverse cognitive deficits by increasing brain cholinergic activity with the use of acetylcholinesterase (AChE) inhibitors, acetylcholine precursors, or cholinergic agonist. The therapeutic strategies for the prevention and/or treatment of cognitive disorder aimed to improve ACh activity. Therefore, the cholinergic receptor agonists (muscarinic and nicotinic) and enhancer of endogenous levels of ACh have been used in patients with Alzheimer-type senile dementia [17–19]. Based on this result, the commonly prescribed AChE inhibitors, such as donepezil, rivastigmine, and galantamine, have been developed. Although AChE inhibitors can diminish the severity of AD, their effects on memory function is still not verified and elucidated.

First, to induce learning and memory impairment, we have intraperitoneally injected SCO in C57BL/6 mice and performed Y-maze, Morris water maze, passive avoidance, and contextual fear conditioning tests. Numerous studies reported the compromised cognitive performance after systemic treatment with SCO, for example, deficits in contextual and cued fear conditioning, impairments in inhibitory avoidance, and alterations in short-term and long-term spatial learning [20, 21].

In the Y-maze task, SCO-treated mice showed decreased spontaneous alteration compared with the vehicle-treated mice, which was increased by FGE and GIN pretreatments. In the Morris water maze task, the SCO-treated mice took a longer time to find the platform than the sham control group, and the FGE- or GIN- and SCO-treated group in combination easily found the location of the hidden platform. In the passive avoidance and contextual fear conditioning tests, SCO treatment reduced the step-through latency as well as freezing time, which were effectively restored by the daily oral administration of GIN.

To support our findings, the administration of Zingicomb, a mixture of* Zingiber officinale* and* G. biloba* extracts, had been shown to improve inhibitory avoidance learning and spatial learning in old Wistar rats [15]. Naturally occurring phytochemicals, which can restore the cognitive dysfunction caused by SCO, were investigated and considered as promising candidates in other studies.* G. biloba* extract, a well-known neuroprotective natural medicine, has been reported to improve memory impairments in the SCO-treated rodent models [22, 23] and humans with AD [24, 25].* G. biloba* has cholinergic actions, including the modulation of presynaptic choline uptake and acetylcholine release, upregulation of postsynaptic muscarinic receptors, and indirect modulation of the serotonergic system [25].* Huperzine A*, which is another neuroprotective phytochemical candidate with the most selective AChE inhibitory activity, significantly restored memory deficits caused by SCO or the normal aging process as determined by the water maze test [26, 27]. In another study, the alcoholic extract of ginger improved memory impairment and infarct volume caused by focal cerebral ischemia by suppressing oxidative stress and fortifying antioxidant enzymes in Wistar rats [28].

As a molecular mechanism underlying the memory enhancing effect of GIN against SCO-induced amnesia, the expression of BDNF was examined. It has been suggested that neurodegeneration is associated with a lack of neurotrophic support, such as BDNF in cholinergic neurons. BDNF is a family of neurotrophins that interact with high-affinity receptor TrkB and play an important role in neuronal survival and maintenance of different types of neurons, synaptic plasticity, and cognitive processes [29]. The oral administration of GIN significantly upregulated the SCO-reduced protein expression of BDNF in the cortex of C56BL/6 mice, which seemed to be mediated by the activation of CREB via phosphorylation. CREB is a transcription factor with diverse roles in neural functions, such as the regulation of neural plasticity, learning and memory, and neurogenic process and is activated by phosphorylation of upstream kinases, such as ERK1/2 and Akt. In this study, the phosphorylation levels of CREB and its upstream regulator Akt were significantly increased in the cortical regions of GIN-treated mice compared with the SCO-treated amnesic group.

Fermented* Curcuma longa* [30],* Asparagus officinalis* [31],* Aronia melanocarpa* [32], and pine needle [33] extracts improved memory dysfunction in SCO-induced amnesia models by increasing the expression of CREB (or p-CREB) and BDNF. Oleanolic acid is a naturally occurring triterpenoid that ameliorated SCO-induced memory impairment by modulating the BDNF-ERK1/2-CREB pathway [34]. Z-guggulsterone, a compound extracted from the resin of the plant* Commiphora wightii*, improved SCO-induced memory loss through the enhancement of the CREB-BDNF signal by the activation of ERK1/2 as well as Akt via phosphorylation [35].

In summary, GIN improved the SCO-induced learning and memory impairment and cognitive dysfunction in C57BL/6 mice based on the results of diverse behavior tests, such as Y-maze, Morris water maze, passive avoidance, and contextual fear conditioning tests. As a neuroprotective molecular mechanism, the oral administration of GIN upregulated the protein levels of neurotrophic factor BDNF, which seemed to be mediated by the activation of Akt-CREB pathway. These results suggested that GIN may have therapeutic potentials in preventing and/or treating memory loss in individuals with amnesia and neurodegenerative diseases, such as AD. However, further studies should be conducted to elucidate the molecular targets of GIN in diverse brain regions and their cross-talk in cellular signaling cascades.
